# Review: Tauopathy in the retina and optic nerve: does it shadow pathological changes in the brain?

**Published:** 2012-11-12

**Authors:** Wing-Lau Ho, Yen Leung, Andrea Wing-Ting Tsang, Kwok-Fai So, Kin Chiu, Raymond Chuen-Chung Chang

**Affiliations:** 1 Laboratory of Neurodegenerative Diseases, Department of Anatomy, LKS Faculty of Medicine; The University of Hong Kong, Pokfulam, Hong Kong SAR, China; 2 Research Centre of Heart, Brain, Hormone and Healthy Aging, LKS Faculty of Medicine; The University of Hong Kong, Pokfulam, Hong Kong SAR, China; 3 State Key Laboratory of Brain and Cognitive Sciences, The University of Hong Kong, Pokfulam, Hong Kong SAR, China

## Abstract

Tau protein’s versatility lies in its functions within the central nervous system, including protein scaffolding and intracellular signaling. Tauopathy has been one of the most extensively studied neuropathologies among the neurodegenerative diseases. Because the retina and optic nerve are parts of the central nervous system, we hypothesize that tauopathy also plays a role in various eye diseases. However, little is known about tauopathy in the retina and optic nerve. Here, we summarize the findings from histopathological studies on animal models and human specimens with distinct neurodegenerative diseases. Similar pathological changes of tau protein can be found in Alzheimer’s disease, frontotemporal lobe dementia, and glaucoma. In view of the important roles of tauopathy in the brain, it is hoped that this review can stimulate research on eye diseases of the retina and optic nerve.

## NEURODEGENERATION AND THE RETINA

Neurodegeneration is a collective term for the progressive loss of the structure, function, or even death of neurons. The pathogenesis of neurodegeneration involves the aberrant processing, misfolding, and subsequent aggregation of normal proteins that are then deposited both intracellularly and extracellularly. At the molecular level, it involves mechanisms such as defective neuronal plasticity through aberrant regenerative responses, reduced levels of neurotrophins, and increased neuronal vulnerability to stress. At least three types of abnormally aggregated proteins have been found. These include β-amyloid (Aβ) peptides in the neuritic plaques of Alzheimer’s disease (AD), the fibrillation of tau protein to form neurofibrillary tangles in AD and Pick bodies in frontotemporal dementia (FTD), and alpha-synuclein in the Lewy bodies of Parkinson disease (PD) and in Lewy body disease [1,2].

The retina is part of the central nervous system (CNS), with various cell types comprising of photoreceptors, bipolar cells, horizontal cells, amacrine cells, and retinal ganglion cells (RGCs). The axons of RGCs forming the last stop of the relay merge to form the optic nerve, optic chiasm, and optic tract, which ultimately forms synapses at the lateral geniculate nucleus of the thalamus. Therefore, the retina and optic nerve could be affected by similar degenerative processes during neurodegeneration.

Evidence suggests that the visual system is affected by neurodegenerative processes in AD. Cognitive visual changes have been reported in patients in the early stages of AD, including difficulties in reading and finding objects [3-5], depth perception, perceiving structure from motion [3,5-7], color recognition [3,8], and impairment of spatial contrast sensitivity [6,9]. Previously, all these defects were considered to be due to pathological changes in the cortex. With the development of modern ocular imaging techniques, recent findings demonstrate that pathological changes within the retina could be found in these patients. Optic coherent tomography has demonstrated a significant reduction of peripapillary retinal fiber layer (RFL) thickness in patients with early AD when compared with age-matched controls [10-14]. The thinning of the RFL was observed predominantly in the inferior and superior quadrants, which was consistent with the inferior and superior visual field loss in AD [3,11]. Reduction of the macular thickness has also been reported in AD, and the total volume of the macula was inversely correlated with the severity of the disease [12]. Changes in the optic nerve head have been observed, using confocal scanning laser ophthalmoscopy. The observed changes included reduced RFL thickness, neuroretinal rim volume and area, and an increased cup–disc ratio, suggesting an overall reduction in the number of optic nerve fibers passing through the optic nerve head [15]. Moreover, laser Doppler flowmetry has demonstrated that retinal blood-flow rate is reduced in AD patients [11].

Histological analysis of postmortem AD samples showed prominent retinal defects, including axonal degeneration in optic nerves, reduced thickness of the RFL, and a significant reduction in the number of large-diameter RGCs [16,17]. Structural analysis of the retina revealed signs of intracellular injuries in these RGCs, ranging from pale cytoplasm with swollen mitochondria and endoplasmic reticulum, to pale nuclei with dispersed chromatin at early stages, to vacuolated cytoplasm and clumped chromatin at late stages [18]. Apoptosis of RGCs and astrocytosis have been observed in AD animal models [19]. These pathological changes are largely attributed to perturbations of amyloid precursor proteins and their processing [19-21]. However, with increasing knowledge regarding tau neurotoxicity, the possible role of tau in retinal pathogenesis should not be neglected. Tau is known to play a pivotal role in multiple neurologic pathways, and it has been shown to interact with signaling pathways that regulate microtubule stability and axonal transport. In addition, tau may bridge various signaling protein complexes during disease progression in the retina [22].

## TAU PROTEINS

Tau proteins are most abundantly found in neurons and astrocytes within the CNS. They are derived from the gene known as microtubule-associated protein tau, on chromosome 17 at position 17q21. This gene contains 16 exons in total; 11 of which are expressed in the human brain. Exons 2, 3, and 10 of the gene are alternatively spliced to produce six isoforms of tau. Each isoform differs in the N-terminus by the number of copies of a 29-amino acid repeat (0N, 1N, or 2N), and in the C-terminus by having either three microtubule binding repeats (3R tau) or four microtubule binding repeats (4R tau) [23].

## Functions of tau proteins

Tau proteins are important in the stabilization and assembly of microtubules, and in turn, they affect the intraneuronal transport of cargos. Thus, under aberrant conditions, the dysregulation of tau proteins may lead to dysfunctional axonal transport and even retraction of spines [2]. Tau may also be involved in signaling pathways by interacting with actin via acidic N-terminals, projecting from microtubules for neurite outgrowth and stabilization during brain development [24]. Different isoforms are distributed unevenly in the neuronal subpopulation, suggesting that there may be specific functions for each isoform.

Apart from the microtubule-associated functions of tau, recent studies have suggested that tau may have a more versatile role in the CNS. First, tau may act as protein scaffolding, and subsequent regulation of its binding partners may affect signaling pathways, such as the Src family kinases [25]. Tau also regulates neurite extension, possibly through its ability to stop microtubule-severing proteins and its facilitative role in nerve growth-factor signaling [26]. Recent findings also reported the capacity of tau to modulate phospholipase C gamma [27], histone deacetylase-6 [28], and heat-shock proteins [29]. Moreover, it has been found that neurogenesis may be severely reduced in tau knockout mice [30].

The phosphorylation state of tau alters its intrinsic functions and binding affinity to microtubules. Hyperphosphorylated tau proteins will aggregate into oligomers and fibrils, and then form neurofibrillary tangles in the somatodendritic compartments of neurons [22].

## Tauopathy

Tauopathy has been found in many neurological disorders, such as posttraumatic degeneration, infections, metabolic diseases, and motor neuron degeneration. The spatial distribution, temporal appearance, and structural changes of tau proteins manifest differently among various neurodegenerative diseases. AD patients have twisted, hyperphosphorylated, and single nonperiodical tau filaments, whereas patients having progressive supernuclear palsy and FTD tend to have only straight tau filaments [31].

## Transgenic mouse models of tauopathy

Most of these mutations cluster in the portion encoding the C-terminal region or in an intervening sequence near exon 10 (Goedert et al., 2000) [32]. Owing to alternative splicing of exons, missense mutations found within exon 10 affect only the three 4R isoforms, whereas those found outside this region affect all six tau isoforms [32].The tau gene has 16 exons. More than 42 mutations associated with FTD-related disorders have been identified, including missense mutations and mutations that affect splicing. Most of these mutations cluster in the portion encoding the C-terminal region or in an intervening sequence near exon 10. The missense mutations impair the binding of tau to microtubules. Apart from that, most of the silent mutations increase the 4R/3R ratio by modulating alternative splicing of exon 10. Owing to alternative splicing of exons, missense mutations found within exon 10 affect only the three 4R isoforms, whereas those found outside this region affect all six tau isoforms.

Differences in the clinical and pathological phenotypes have been observed between various tau mutations. Although many mutations result in a phenotype resembling FTD, others may lead to phenotypes overlapping or identical to Pick’s disease, corticobasal degeneration, progressive supernuclear palsy, or even AD. Some mutations lead to tau pathology in both neurons and glial cells, whereas others lead to tau pathology mainly in neurons. These mutations may result in tau filaments and may produce paired helical filaments (PHF) or straight filaments. Furthermore, the same mutation may have several phenotypic presentations if different promoters are used to drive their expressions.

A commonly used transgenic mouse model of tauopathy is human mutated tau P301L. The first strain with P301L was the JNPL3 line bearing the 4R/0N tau gene driven by mouse prion promoter. P301L mutation is the conversion of proline residue 301 to leucine. JNPL3 mice are a strain of transgenic mice expressing the 4R/0N isoform of tau bearing the P301L mutation. As defined in the previous paragraph, 4R/0N stands for the tau isoform bearing 4 microtubule binding repeats and no copies of the 29-amino acid repeat in the N-terminus. Histologically, the mice develop neurofibrillary tangles and Pick-body-like lesions in the amygdala, septal nuclei, preoptic nuclei, hypothalamus, midbrain, pons, medulla, deep cerebellar nuclei, and spinal cord. Aside from neurons, glial cells were also found to contain tau filaments. Axonal degeneration has been observed in the spinal cord, leading to progressive loss of motor neurons [33,34]. Neurons undergoing neurofibrillary degeneration exhibit tau immunoreactivity, mainly in 15–20 nm-wide, straight or wavy filaments with no periodic twists. The filaments push away the intracellular organelles. In addition, the nuclei appear to be irregular and lobulated due to deep membrane folding. Golgi complexes are dilated and vacuolated, suggesting active cellular activities [35]. The surrounding oligodendrocytes are also affected by axonal degeneration. Reactive astrocytosis can be observed at as early as 2 months of age [36,37]. The JNPL3 strains show age- and sex-dependent production of tau, with an increase in phosphorylation of insoluble tau as the mice age. There is a higher tau–tubulin ratio and more insoluble tau in the subcortical region compared to the corticolimbal region [38].

The pR5 mouse is another tau transgenic mouse model bearing the P301L mutation. Under control of the neuron-specific mouse glucophosphatidylinositol linked protein 1.2 (mThy1.2) promoter, the pR5 line overexpresses the longest human tau isoform (2^+^3^+^4R) together with P301L. The pR5 model has been used to study the interaction of Aβ peptide and tau. Injection of Aβ peptide in this strain resulted in the stress response of proteosomal degradation of misfolded proteins, together with upregulation of nucleotide metabolism and transcription of proteins. In addition, energy metabolism was affected due to mitochondrial dysfunction. The disruptive effect of Aβ peptide on synapse-related proteins, cytoskeletal organization, biogenesis of related proteins, and intracellular pH regulation suggests that the effect of Aβ peptide in neuronal toxicity is augmented by tau [39].

A third strain of transgenic mice with the P301L mutation, Tg23027, incorporates the P301L mutation into the longest tau isoform (4R2N) under the control of the hamster promoter. These mice develop neurofibrillary tangles and glial tangle pathology in the frontotemporal region, the brainstem, and to a lesser extent, the spinal cord [40]. The abnormal pathologies of neuronal loss and cerebral atrophy progress with age [40].

A conditional transgenic P301L-mutation mouse model, rTg4510, has also commonly been used. The promoter region contains a forebrain-specific calmodulin kinase II (CaM KII) promoter system that can be switched on by administrating tetracycline. This model has two significant advantages in experimentation. First, this model generates a more prominent expression of abnormal tau in forebrain structures such as the hippocampus and neocortex. Second, regulation of transcription can be achieved, because the gene can be switched on by tetracycline and switched off by doxycycline. Histopathology shows abnormal biochemical processing of tau. Neurofibrillary tangles can be observed in the neocortex, hippocampus, and the limbic structures [41].

In the P301L strains, mitochondrial dysfunctions, particularly in the mitochondrial complex V, have been found to lead to impairment of mitochondrial respiration and adenosine triphosphate (ATP) synthesis. Functional analysis has demonstrated mitochondrial dysfunctions, such as reduced activity of nicotinamide adenine dinucleotide (reduced form):ubiquinone oxidoreductase with increasing in P301L tau mice. Mitochondrial dysfunctions are associated with high levels of reactive oxygen species in aged transgenic mice. Aged homozygous P301L tau mice display modified lipid peroxidation levels and the upregulation of antioxidant enzymes in response to oxidative stress. Furthermore, mitochondria in P301L tau mice are more vulnerable to Aβ peptide toxicity, suggesting the synergistic action of tau and Aβ peptide pathology on the mitochondria [42].

P301S is another widely used tau mutation, with one 4R0N driven by Thy1.2 promoter. The mice exhibit neuroinflammation as well as hyperphosphorylation of tau, with tangle formation in the brain and spinal cord, resembling tau aggregates found in FTDP-17. The majority of filaments resemble the half-twisted ribbons described previously in FTDP-17, with a minority of filaments resembling the PHF-tau in AD.

Another P301S with the 4R1N strain shows loss of synapses and activation of microglia by 3–6 months in the hippocampus. In older mice of this strain, accumulation of tau has been found in parallel with neuronal loss and atrophy of the hippocampus [43,44].

In spite of the above phenotypes, the transgenic mice bearing the P301S mutation do not always display severe motor deficits. Schinkdowski and colleagues [45] created a mouse line bearing two tau gene mutations, P301S and G272V. The mice did not display motor dysfunction; instead, they demonstrated increased anxiety and impairments of spatial memory. Extensive neurofibrillary tangles were found in the amygdala and the hippocampus, with rare ghost tangles and PHF-like changes in addition to mild astrogliosis. The hippocampus was also found to have progressive cell loss and changes in synaptic transmission by 14 months of age. Behavioral impairment related to hippocampus disorder was characterized by increased anxiety, delayed learning from 3 months of age, and reduced spatial memory at 10 months of age.

## Tau and the retina

**Figure 1:**
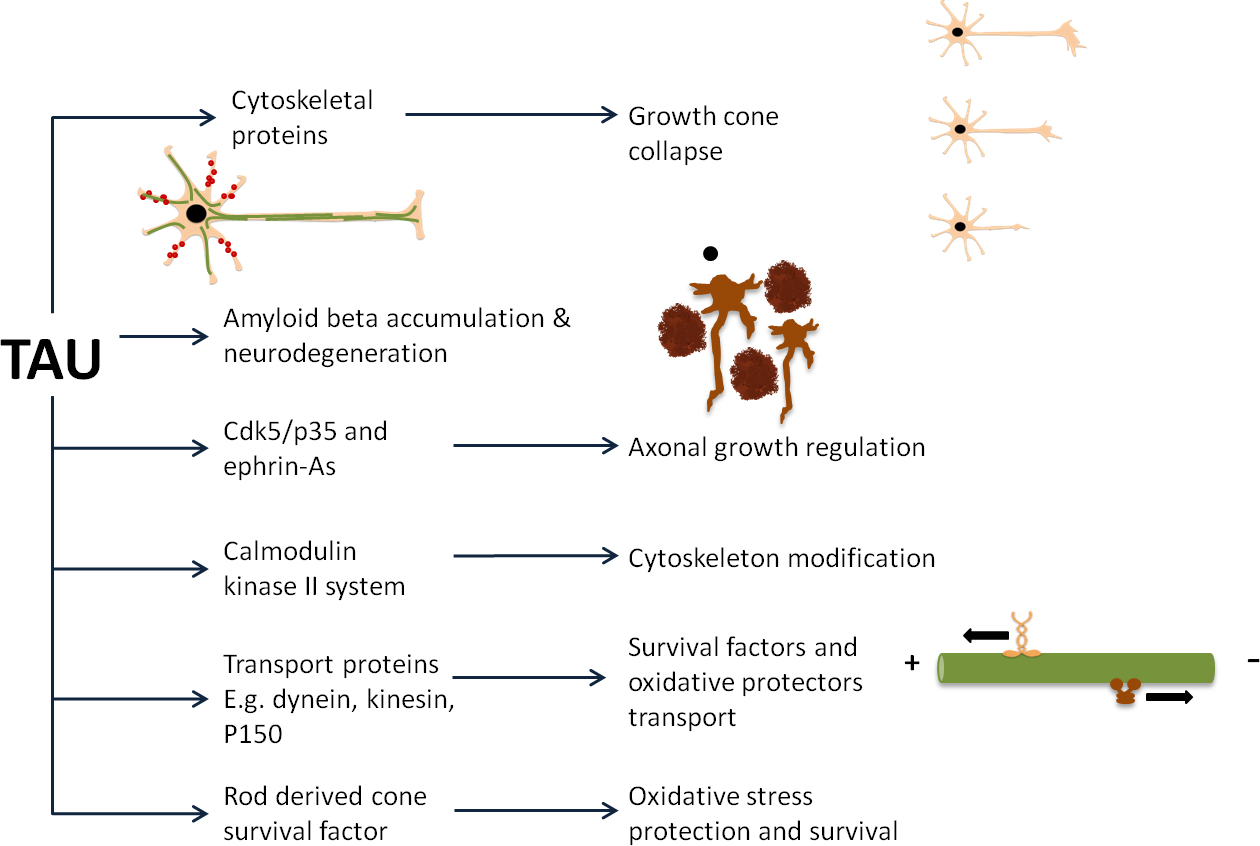
Schematic diagram summarizing the roles of tau in retinal functions. Tau can stabilize microtubules. Any distortion of tau protein leading it leaving microtubules can result in growth cone collapse. Beta-amyloid (Aβ) is another example to trigger phosphorylation of tau. As a result, tau departs from microtubule resulting in neurodegeneration. Apart from Aβ as a triggering factor, any stimulation of signaling cascade of cyclin-dependent kinase 5 (cdk5), Eph family receptor interacting protein A (ephrin-A) receptor, or calcium/calmodulin dependent protein kinase II (CaM KII) affecting phosphorylation of tau can also affect microtubules. Once tau leaves microtubules after phosphorylation, they can easily form aggregation, which can further impair axonal transport mediated by kinesin or dynein. Consequently, mitochondria in the distal part of nerve, nerve terminal or spines cannot obtain protection from the cell body (soma) so that they are collapse and cannot produce energy. Neurodegeneration can unavoidably occur. Tau also interacts with rod-derived viability factor that can inhibit phosphorylation of tau.

Similar to its function in neurons in the brain, tau not only regulates the cytoskeletal and axonal transport in retinal neurons, but also affects Aβ accumulation and cell-survival signaling in the retina. The pivotal roles of tau in retinal functions are summarized in Figure 1.

It has been found that tau is expressed in a gradient manner in RGCs, with higher levels in the terminal parts of axons of developing RGCs. Its localization at the axon plays a role in proper axon development and the survival of RGCs [46]. As mentioned, tau is a cytoskeletal protein that exhibits high affinity for microtubules. Therefore, tau is required for the development of axons. Exposure to okadaic acid has resulted in the accumulation of phosphorylated tau, closely followed by distortion of the cytoskeleton, leading to growth-cone collapse. Tau has hence been implicated in the process of establishing neuronal axon polarity [47].

One of the possible consequences of an interruption in these transport mechanisms would be the accumulation of Aβ, which can propagate secondary degeneration. Studies based on Tg2576 transgenic mice showed that the immunoreactivity of hyperphosphorylated tau was similar to that of Aβ in mouse retinas [48].

Tau can be phosphorylated by cyclin-dependent kinase 5 (Cdk5). As a result, its binding affinity to microtubules is altered. Cdk5 is a proline-directed serine/threonine kinase, and has been shown to phosphorylate tau at several serine-proline and threonine-proline motifs. Phosphorylation at these residues causes tau to dissociate from microtubules, and this has been suggested as affecting microtubule stability. Unlike its kinase family members, Cdk5 does not act to regulate cell cycle progression. Instead, it is highly expressed in neuronal axons and growth cones serving to promote neurite outgrowth and migration [49]. To initiate its activation, Cdk5 requires interaction with its activator subunit p35. Cdk5 regulates a diverse range of cellular processes in the CNS through phosphorylation of various substrates. Studies have shown that the ephrin-A signaling pathway can also lead to the activation of Cdk5. Ephrin-A regulates retinotectal projection by mediating receptor-mediated axon growth repulsion through a complex signaling cascade. Upon the binding of ephrin-A to ephrin-A tyrosine kinase receptors, exchange factors are activated, and subsequently, ras homolog A (RhoA) and Fyn (Fyn is a membrane-associated tyrosine kinase involved in the phosphorylation of tyrosine residues on specific target proteins) are recruited to the receptors. RhoA leads to the activation of Rho-associated kinase (ROCK), which in turn, activates LIM (LIM domains were first discovered in proteins: Lin11, Isl-1 & Mec-3)-kinases to phosphorylate two target proteins destrin and cofilin to promote actin depolymerisation [50]. Concurrently, Fyn activates Cdk5 to phosphorylate collapsin response-mediator protein, to reduce microtubule assembly [51]. Taken together, the stimulation of ephrin-A leads to growth-cone collapse, which occurs largely through vast cytoskeletal rearrangement. Immunofluorescence studies have shown that Cdk5 activation occurs downstream of ephrin-A5 signaling, to phosphorylate tau in the growth cones and axons of RGCs. These findings suggest that phosphorylation of tau serves as another means by which ephrin-A signaling can induce microtubule reorganization in RGC growth cones [52].

Apart from Cdk5, tau has also been found to interact with CaM KII in the CaM KII-α-associated protein complex in the chick retina. Endogenous association of tau with CaM KII-α suggests that it is important in regulating cytoskeletal assembly in neurons. Through the phosphorylation of tau, microtubule assembly may be inhibited, thereby disrupting the cellular architecture [53].

Studies of the functional impact of abnormally aggregated tau, based on rat retina models, have also shown interesting results. In rat RGCs, it has been found that the transportation of mitochondria by kinesin-like motors toward the cell periphery is inhibited by abnormally aggregated tau. Consequently, neurons with perturbation of mitochondria and peroxisomes suffer from loss of energy production and the accumulation of reactive oxygen species. The anterograde transport of vesicles necessary for growth cones and synapse function is slowed down. In addition, these neurons may be more susceptible to oxidative stress [54].

In addition to kinesin, tau can also interact with other proteins in the RGCs. In the RGCs axons of P301S mutant mice, the projection domain of tau interacts with the C-terminus of P150, the major component of the dynein activator. The co-localization of tau and P150 suggests that tau dysfunction can result in the mislocalization of dynactin in axons, which can result in neurodegeneration [55].

Tau may also interact with other factors. Rod-derived cone viability factor is responsible for maintaining the function and subsequently the viability of photoreceptor cells. In a mouse model of retinal degeneration, mutant mice carrying null mutations have been found to exhibit an accumulation of hyperphosphorylated tau. A degeneration pattern of rod photoreceptors in these mice has been found in regions coinciding with the aggregation of hyperphosphorylated tau in the retina. It has been suggested that rod-derived cone viability factor inhibits phosphorylation and oxidation of tau, and hence may have a therapeutic role [56].

## Tau in ocular disease

**Figure 2:**
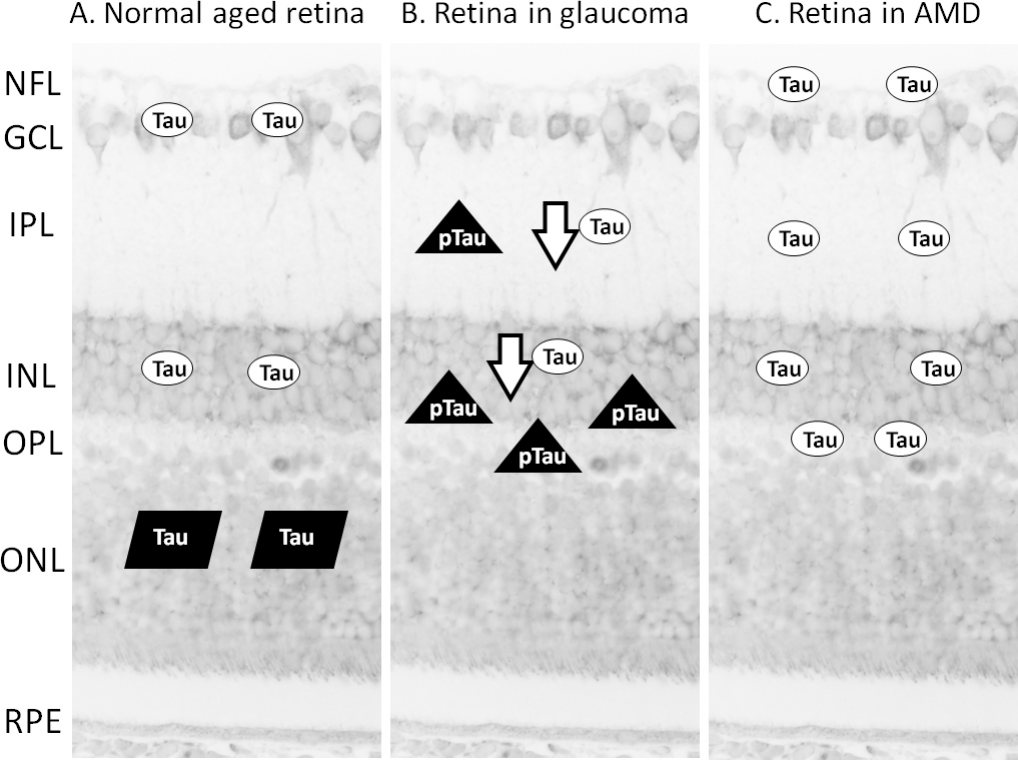
Diagram summarizing the literature reporting on the distribution of tau in the retina of normal and pathological states. The background is a cross section showing the layered structure of the human retina. Ovals labeled Tau represent expression of total tau. Parallelograms labeled Tau represent tau aggregates. Triangles labeled pTau represent expression of abnormal phosphorylated tau. **A**: The distribution of tau in the normal aged retina is illustrated. **B**: The distribution of tau in the glaucomatous retina is illustrated. **C**: The distribution of tau in the retina in age-related macular degeneration (AMD) is illustrated. Abbreviations: nerve fiber layer (NFL), ganglion cell layer (GCL), inner plexiform layer (IPL), inner nuclear layer (INL), outer plexiform layer (OPL), outer nuclear layer (ONL), retinal pigment epithelium (RPE).

In a study based on immunohistochemical analysis of 19 nucleated eyes from patients aged 49–87 years, diffuse immunoreactivity of tau was found in the inner nuclear layer in all patients, while it was found in seven cases in RGCs [57]. The study established a positive correlation between age and number of tau-positive RGCs [57]. In addition, aggregated tau was found within the cytoplasm of photoreceptor cells in nine patients older than 63 years [57]. Although another study did not show variation of staining intensity with age or disease status among patients with age-related macular degeneration (AMD) and retinitis pigmentosa, it did confirm that the immunoreactivity of tau could be detected in the inner nuclear layer, inner plexiform layer, outer plexiform layer, and nerve fiber layer [58]. Figure 2A summarizes the distribution of tau in the aged retina.

Analysis of vitretomized samples demonstrated that a significant decrease in Aβ peptide and an increase in tau protein occurred in diabetic retinopathy and other ocular diseases [59]. It has been suggested that Aβ deposition and tau hyperphosphorylation may be partly involved in some of the degenerative diseases of the retina and that the pathology may be similar to that observed in the brain [59].

Specimens from patients with glaucoma have also shown the presence of tau. A human case-control analysis of 11 surgical glaucomatous eyes and ten age-matched control eyes revealed that normal tau is present in the inner nuclear and inner plexiform layers, but much reduced in glaucomatous retinas [60]. On the other hand, AT8-tau immunoreactivity was found in the glaucomatous retina at the outer border of the inner nuclear layer and occasionally in the inner plexiform layer [60]. However, this pattern was not exhibited in six specimens from patients with incidental open-angle glaucoma. In addition, abnormal tau was also found in horizontal cells based on the study of co-localization by a horizontal cell marker, parvalbumin [60]. The distribution of tau in the glaucomatous retina is summarized in Figure 2B. All these findings support the hypothesis that glaucoma shares pathways with other neurodegenerative diseases.

This is consistent with previous reports showing an increased incidence of primary open-angle glaucoma among AD patients, with most patients diagnosed with the normal-tension form. Moreover, glaucoma seems to progress more rapidly in AD patients. Recent evidence also indicates that altered cerebrospinal fluid (CSF) circulatory dynamics can reduce the clearance of both Aβ and tau, whereas decreased Aβ and increased tau levels in the CSF have been associated with the risk of rapid glaucoma progression. One hypothesis for this association is that altered CSF circulatory dynamics can reduce neurotoxin clearance along the optic nerve in the subarachnoid space, leading to deposition of tau and other toxic molecules, which ultimately results in glaucoma progression [61].

In mouse models of ocular hypertension, the loss of tau proteins in the retina has been shown to occur from as early as 4 h to over 7 d after the induction of ocular hypertensive stress [62]. Proteolysis of tau has been suggested as contributing to the pathogenesis of neuronal cell death, correlating with an increase in calcium, which in turn activates calpain, a calcium-dependent protease [62]. The loss of tau is evident even at earlier stages when the outer layer of the retina is mostly intact [62]. This may be attributed to the distribution of tau in the inner retina or may be the effect of other proteases. Phosphorylation of tau has been shown to be involved in retinal cell death as related to ocular hypertension [60]. Calpain-induced conversion of p35 to p25 and activation of Cdk5 are also involved [62]. Studies have not been able to show a direct increase of phosphorylated tau; however, it is indirectly evident from the upregulation of the relevant kinase, Cdk5, and the regulatory protein, p35/p25. One justification for the failure to detect phosphorylated tau is that tau protein is cleaved by calpain before detection is possible [62]. Under hypoxic conditions, similar changes have also been reported. In rat retinas treated with hypoxic conditions, it has been found that the immunoreactivity of tau is almost completely lost in retinas within 5 h; however, the proteolytic products of tau remain detectable [63].

The role of tauopathy in AMD cannot be underestimated. Drusens have been found in dry AMD with a heterogeneous content. Although there was no direct evidence showing that tau is one of the components of drusens, tauopathy and drusens have both been found in aged retina [64]. The distribution of tau in the AMD retina is illustrated in Figure 2C. With the pivotal role of tau in cellular transport, we postulate that defective transportation related to tauopathy may play a role in the deposition of drusens, which may subsequently result in AMD.

Transgenic mouse models have also provided insight into retinal changes in tauopathy. In P301S mice, tau is expressed in RGCs and is phosphorylated in pathological states. Filamentous inclusions in the retina are formed, similar to those found in the brain. However, these pathological changes do not necessarily lead to a decrease in cell number, but rather present as axonal pathology in a punctated morphology. Although this is not the most commonly studied transgenic model, these findings have already pointed out that similar neurodegenerative changes do occur in the retina and the brain [65].

## Future perspectives

Studying the changes in the visual pathways of patients with neurodegeneration is an emerging, yet difficult, research field. Patients with neurodegenerative diseases have difficulty providing satisfactory responses in tests of visual functions. They may not be compliant during these tests, even when the state-of-the-art equipment is used. In addition, postmortem specimens have been difficult to obtain. However, with the development of new research tools such as cell culture techniques, transgenic mouse models, and reliable imaging systems, more attention is being directed toward this area, and progress is currently rewarding.

The amyloid models of neurodegeneration have long been studied in great detail. For those who are interested in studying the interaction between tau, neurodegeneration, and the retina, it would be useful to learn from the experience of amyloid precursor protein models. The interaction between tau and Aβ is complex. Tau can affect the transport of Aβ, and therefore produce Aβ peptide-related pathological events. In addition, tau per se may actually play an important role in neurodegeneration. Furthermore, the combined toxicities of tau and Aβ peptide may then collectively contribute to the final degenerative pathway. The experience of Aβ experimentation can be used in the study of tauopathy.

Tau-related changes in the retina have several important implications. As discussed, there are strengthening lines of evidence suggesting that neurodegenerative processes are similar in ocular diseases and neurodegenerative diseases, such as glaucoma and AD. These diseases share remarkable similarities in visual functions in terms of visual field testing and retinal nerve-fiber thinning [66]. It has been hypothesized that similar apoptotic changes may occur in both diseases due to loss of noradrenaline innervation. Application of alpha-2 adrenergic receptor agonists could demonstrate neuroprotection in both diseases [67]. A study on glaucomatous neuropathy show that RGC axon degeneration occurs before soma death [68]. Transgenic mouse models have shown that axonal degeneration may occur without any influence of pro-apoptotic Bax protein, revealing that differential degeneration processes of RGC occur during glaucoma. Another transgenic mouse model has shown that the slow Wallerian degeneration (WldS) allele slows or prevents RGC axon degeneration in glaucoma. Together, these support the idea that axonal degeneration is an important event in glaucomatous neurodegeneration [69]. Tau has been demonstrated to play a pivotal role in neurodegeneration in pathological eyes. Targeting tau with neuroprotective treatments may not only provide a new therapeutic approach, but also allow us to better understand the pathological mechanisms of tauopathy in neurodegenerative diseases.

It is never easy to assess the CNS in a noninvasive manner, even though magnetic resonance imaging has become common in many hospitals. For ophthalmology, there are many noninvasive tests for the eyes, including optical coherent tomography, electrophysiology testing, and fluorescein angiography. If we can prove that the pathological changes in the eyes actually reflect the changes in the brain parenchyma, monitoring neurodegeneration in the brain can be a simple task. Apart from being useful for studying neurodegeneration, the eyes allow convenient assessment of treatment responses, owing to their ease of access from the outside. In essence, the eyes, in particular, changes in the retina, can provide biomarkers of the pathological changes in the brain.

Studying tauopathy may result in a paradigm shift in the management of ocular diseases. Our understanding of the pathogenesis of some commonly found ocular diseases such as glaucoma and AMD may be altered, which may lead to the development of new therapeutic agents. Ocular screening programs can be launched to offer patients diagnoses of tauopathies such as AD; furthermore, patients with glaucoma may require checkups for central neurodegeneration. Similarly, patients with tau mutations may be under close surveillance for the development of both ocular and systemic diseases. Intraocular and systemic therapeutic agents may be developed, serving the dual purposes of treating both central and ocular conditions.
